# Multiple Modes of Action of Anti-TNF-α Antibodies for Inflammatory Bowel Diseases Beyond TNF-α Neutralization

**DOI:** 10.3390/antib15040071

**Published:** 2026-08-06

**Authors:** Tomohiro Watanabe, Masatoshi Kudo

**Affiliations:** Department of Gastroenterology and Hepatology, Faculty of Medicine, Kindai University, 1-14-1 Mihara-dai, Minami-ku, Sakai 590-0197, Japan; m-kudo@med.kindai.ac.jp

**Keywords:** adalimumab, apoptosis, golimumab, inflammatory bowel disease, infliximab, regulatory T cell, TNF-α

## Abstract

Inflammatory bowel diseases (IBDs), chronic inflammatory disorders of the gastrointestinal tract, are classified into Crohn’s disease (CD) and ulcerative colitis (UC). Despite differing clinical manifestations, both CD and UC share a common immunopathogenesis, involving excessive production of proinflammatory cytokines by macrophages and dendritic cells. Tumor necrosis factor-α (TNF-α), interleukin-12 (IL-12), and IL-23 are prototypical proinflammatory cytokines associated with CD and UC development, and biologics targeting these cytokines are widely used for treating patients with IBD. Antibodies (Abs) against IL-12 and IL-23 decrease intestinal inflammation by binding to the soluble forms of IL-12 and IL-23, respectively. Notably, anti-TNF-α Abs induce remission by neutralizing soluble TNF-α as well as membrane-bound TNF-α (mTNF-α). The binding of anti-TNF-α Abs to mTNF-α enables the diverse functions of IBD-specific TNF-α-inhibitors. Multiple modes of action of anti-TNF-α Abs for IBD include complement-dependent cytotoxicity, Ab-dependent cellular cytotoxicity, apoptosis of cells expressing mTNF-α, apoptosis of lamina propria T cells, and induction of regulatory T cells and macrophages. In this article, we introduce the diverse actions of anti-TNF-α Abs beyond TNF-α neutralization and discuss how these unique properties of anti-TNF-α Abs contribute to the efficient downregulation of IBD-associated inflammation.

## 1. Introduction

Crohn’s disease (CD) and ulcerative colitis (UC) are two major forms of inflammatory bowel disease (IBD) [1,2,3]. CD and UC have distinct clinical features. CD is characterized by skip lesions throughout the gastrointestinal (GI) tract, longitudinal ulcers, and transmural inflammation, whereas UC is diagnosed based on continuous lesions beginning from the rectum to the proximal GI tract, various forms of ulcers and erosions, and superficial inflammation [1,2,3]. Despite these differences in clinical manifestations, CD and UC share a common immunopathogenesis as both disorders are driven by proinflammatory cytokine responses against intestinal microbiota [1,2,3].

Tumor necrosis factor-α (TNF-α), interleukin (IL)-1β, IL-6, IL-12, and IL-23 are the prototypical proinflammatory cytokines in IBD [1,2,3]. Evidence supporting this idea comes from the marked efficacy and safety of biologics targeting TNF-α, IL-12, and IL-23 in patients with IBD [1,2]. Biologics targeting TNF-α were the first ones developed for treating IBD. Adalimumab (ADA), golimumab (GLM), and infliximab (IFX) are IgG1-type monoclonal antibodies (Abs) against human TNF-α and have been successfully introduced for patients with IBD [4]. The efficacy and safety of ADA, GLM, and IFX have been well established and anti-TNF-α Abs have been the first choice for treating IBD resistant to 5 aminosalicylic acid and prednisolone (PSL). However, the recent development of biologics targeting IL-12, IL-23, or janus kinase inhibitors obligates the IBD clinicians to consider biologics or small molecules targeting cytokines other than TNF-α as priority drugs in some cases. However, anti-TNF-α Abs for IBD notably exert their immunoregulatory actions through binding not only to soluble TNF-α (sTNF-α) but also to membrane-bound TNF-α (mTNF-α). On the contrary, anti-IL-12/IL-23p40 Ab and anti-IL-23p19 Ab only neutralize the soluble forms of IL-12/23p40 and IL-23p19, respectively. Anti-TNF-α Abs for IBD have diverse functions in addition to the blockade of TNF-α-mediated signaling pathways. In this review, we introduce the multiple modes of action of anti-TNF-α Abs (ADA, GLM, and IFX) and discuss how the unique properties of these biologics mediate the downregulation of chronic GI inflammation.

## 2. Pathogenicity of mTNF-α in IBD

TNF-α is produced as mTNF-α, which is subjected to cleavage by TNF-α-converting enzyme, and then secreted as sTNF-α [5]. sTNF-α and mTNF-α bind to TNF receptor 1 (TNFR1) and TNFR2, both of which are expressed on various cell types, including T cells, B cells, macrophages, DCs, and epithelial cells [5]. TNF-α binding to TNFR1 and TNFR2 induces proinflammatory cytokine production through the nuclear translocation of nuclear factor-κB (NF-κB) subunits. NF-κB-dependent proinflammatory cytokines include IL-1β, IL-6, IL-12, IL-23, and TNF-α [1,2,3]. In addition to enhancing proinflammatory cytokine responses, TNF-α binding to TNFR1 expressed on epithelial cells directly induces epithelial injury through necroptosis [5]. Epithelial cell death induced by TNF-α-mediated necroptosis impairs the intestinal barrier, allowing invasion of intestinal microbiota into the lamina propria, and then causes excessive production of proinflammatory cytokines by macrophages and DCs upon exposure to the bacteria [6]. Thus, TNF-α plays central roles in the formation of colitogenic immune environments in the GI tract.

Immunofluorescence studies have demonstrated TNF-α expression on the membranes of immune cells in the inflamed GI mucosa of patients with IBD [7]. In collaboration with human studies, Corazza et al. reported that mTNF-α expression is sufficient for developing T cell transfer colitis and that colitis can occur even in the absence of sTNF-α [8]. These studies suggest that rather than sTNF-α, mTNF-α plays pathogenic roles in human and murine IBD. This notion is fully supported by the results of clinical trials; ADA, GLM, and IFX, all of which are biologics that bind to both mTNF-α and sTNF-α, are effective at inducing and maintaining remission in patients with IBD, whereas etanercept (ETN), which lacks neutralizing activity against mTNF-α, fails to induce remission in IBD [1,2,3,9]. Importantly, the anti-TNF-α Abs used for treating IBD have multiple modes of action for inducing and maintaining remission beyond the neutralization of TNF-α-mediated signaling pathways.

## 3. Multiple Modes of Action of Anti-TNF-α Abs

**Figure 1:**
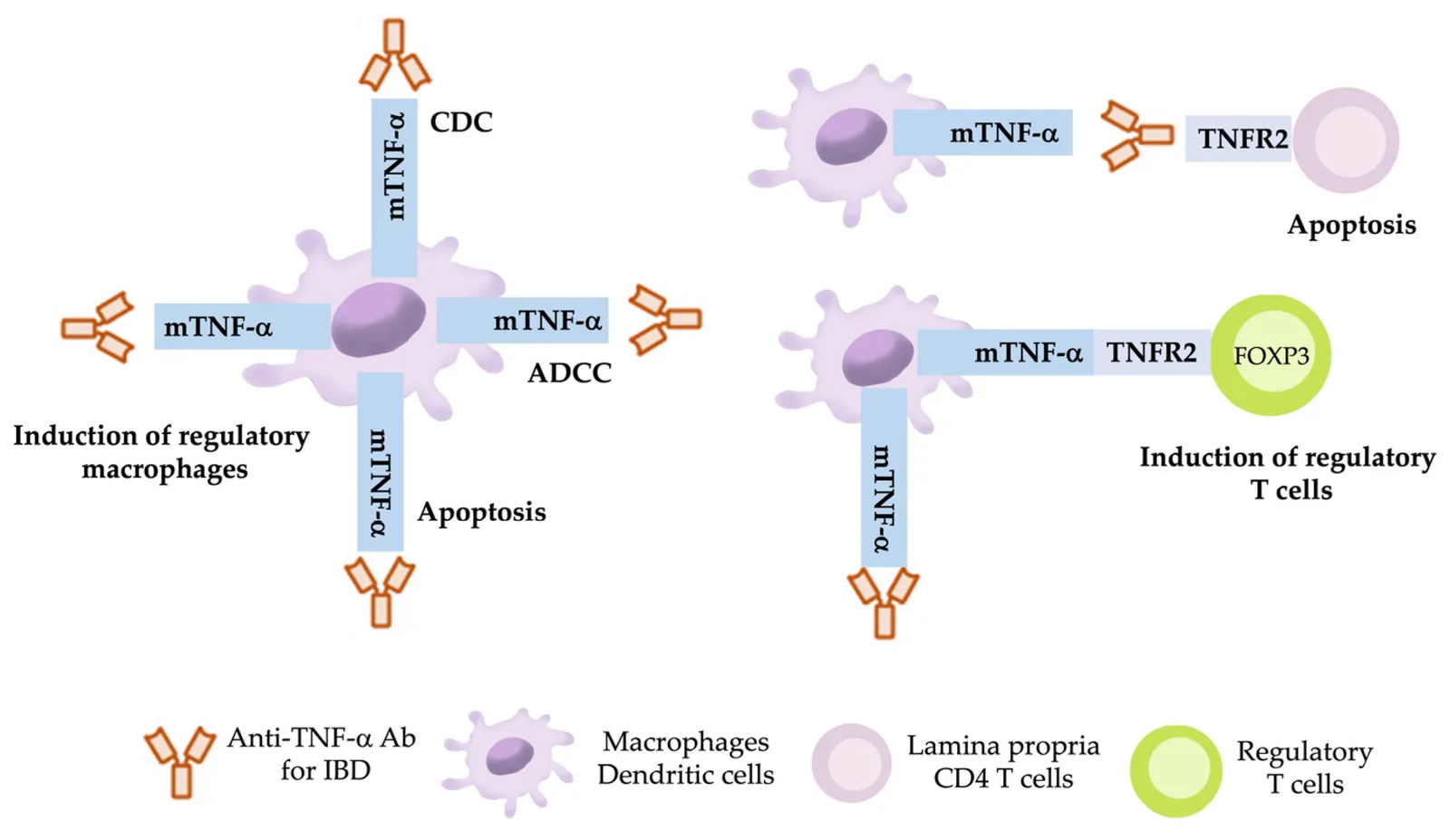
**Multiple modes of action by anti-TNF-α antibodies used for IBD.** Six mechanisms of action of anti-TNF-α antibodies are shown. Abbreviations: ADCC; antibody-dependent cellular cytotoxicity, CDC; complement-dependent cytotoxicity, FOXP3; forkhead box p3; IBD; inflammatory bowel disease, mTNF-α; membrane-bound TNF-α, TNFR2; tumor necrosis factor receptor 2.

ADA, GLM, and IFX are widely used for treating IBD in Japan, and the safety and efficacy of these biologics with the ability to neutralize mTNF-α and sTNF-α are well-established [1,2]. In this section, we summarize the molecular mechanisms by which treatment with ADA, GLM, and IFX induces remission in patients with IBD via binding to mTNF-α (Figure 1 and Table 1). Pharmacokinetic data are shown in Table 1 [10].

### 3.1. Complement-Dependent Cytotoxicity Against Cells Expressing mTNF-α

Cell death caused by cytotoxicity is a major mechanism underlying the efficient control of proinflammatory responses by ADA, GLM, and IFX. Previous studies have addressed whether anti-TNF-α exerts complement-dependent cytotoxicity (CDC) activity against cells overexpressing mTNF-α in a cell culture system by utilizing fresh human serum or heat-inactivated serum [11,12,13]. Exposure to ADA, GLM, and IFX at the doses of 1 μg/mL significantly increased the percentages of propidium iodide (PI)-positive cells, whereas ETN failed to induce complement-dependent cell death at the same dose [11,12,13]. The ETN dose required to achieve a significant CDC was 10 μg/mL. CDC activity was comparable between ADA, GLM, and IFX as assessed by the alterations in PI-positive dead cells [11,12,13]. Although the molecular mechanisms accounting for the insufficient CDC activity of ETN, compared with that of ADA, GLM, and IFX, remain to be fully understood, the structural differences in anti-TNF-α Abs are likely to be involved. ETN does not have the constant heavy domain 1 required for complement 3 activation [4]. Overall, these in vitro studies suggest that the death of pathogenic macrophages and DCs expressing mTNF-α in the presence of complements may contribute to efficient induction of IBD remission by ADA, GLM, and IFX.

### 3.2. Antibody-Dependent Cellular Cytotoxicity Against Cells Expressing mTNF-α

Ab-coated cells are recognized and killed by Fc receptor (FcR)-expressing macrophages, DCs, and natural killer cells. This immune process is called Ab-dependent cellular cytotoxicity (ADCC). ADCC activity was examined in vitro using Jurkat T cells overexpressing mTNF-α and human peripheral blood mononuclear cells (PBMCs) as target and effector cells, respectively [11,12]. The percentages of target cell death were comparable when human PBMCs were co-cultured with effector cells in the presence of ADA, ETN, GLM, and IFX. Thus, these studies demonstrate that anti-TNF-α Abs for IBD may reduce colitogenic responses through the death of pathogenic macrophages and DCs expressing mTNF-α by CDC and ADCC.

### 3.3. Apoptosis of mTNF-α-Expressing Cells

In addition to CDC and ADCC, ADA, GLM, and IFX can induce apoptosis in mTNF-α-expressing cells. The percentages of Annexin-V^+^PI^−^ apoptotic cells were significantly increased in Jurkat T cells overexpressing mTNF-α upon adding ADA, GLM, and IFX [11,12]. Apoptotic cell death was not induced by adding ETN. Notably, the percentages of Annexin-V^+^PI^−^ apoptotic cells were greater in cells stimulated with ADA or IFX than in those stimulated with GLM. Mechanistically, three serine residues at the cytoplasmic domain of mTNF-α are required for apoptosis induction by IFX because IFX-induced apoptotic cell death was not observed in Jurkat T cells overexpressing mutant mTNF-α, in which these serine residues were replaced with alanine [14]. Further, IFX binding to mTNF-α induces accumulation of reactive oxygen species and activation of c-JUN N-terminal kinase, thereby causing G0/G1 cell cycle arrest [14]. Thus, binding of ADA and IFX to mTNF-α results in apoptotic cell death of colitogenic macrophages and DCs in the GI tract via outside-to-inside signals. Collectively, pathogenic macrophages and DCs expressing mTNF-α are likely to be eliminated by ADA, GLM, and IFX in the active mucosa of patients with IBD. Consistent with these in vitro studies showing cytotoxic and apoptotic cell death of macrophages and DCs expressing mTNF-α, Caprioli et al. reported that IFX-induced resolution of intestinal inflammation is accompanied by reduction in intestinal CD68^+^ macrophages expressing mTNF-α caused by apoptosis in patients with IBD [15]. Furthermore, induction of IBD remission by IFX is associated with a decrease in IL-23p19-expressing CD68^+^ macrophages [15]. Therefore, cell death of macrophages and DCs expressing both mTNF-α and IL-23p19 may contribute to the suppression of chronic inflammatory responses in the GI mucosa.

### 3.4. Apoptosis of Lamina Propria T Cells

As mentioned above, the binding of ADA, GLM, and IFX to mTNF-α and sTNF-α neutralizes TNF-α-mediated signaling and also kills macrophages and DCs expressing mTNF-α. In addition to cell death in macrophages and DCs, the apoptotic cell death of T cells is induced in the GI mucosa of patients with IBD after administration of ADA and IFX [16,17]. Annexin V^+^CD4^+^ T cells were found to be markedly increased in colonic biopsy specimens obtained from patients with IBD at 24 h after IFX administration [16]. Parallel with this observation, Atreya et al. also reported apoptosis induction in CD4^+^ T cells in the GI mucosa of patients with IBD treated with ADA or IFX [17]. Survival of CD4^+^ T cells in the GI tract requires the interaction between TNFR2 and mTNF-α expressed on CD4^+^ T cells and macrophages, respectively. Blockade of the TNFR2-mTNF-α interaction by ADA or IFX makes CD4^+^TNFR2^+^ T cells susceptible to apoptosis [17]. IL-6 can antagonize anti-TNF-α Ab-induced apoptosis of CD4^+^TNFR2^+^ T cells through activation of signal transduction and activator of transcription 3. Whether induction of apoptosis in CD4^+^TNFR^+^ T cells by these TNF-α-blockers reduces production of colitogenic cytokine responses (IFN-γ and IL-17) by CD4^+^ T cells remains unclear.

### 3.5. Induction of Regulatory T Cells

Regulatory T cells (Tregs) expressing forkhead box p3 (FOXP3) maintain intestinal immune homeostasis by inhibiting proinflammatory cytokine responses against microbiota [2]. Importantly, ADA and IFX induce expansion of Tregs [18,19,20,21]. Apoptosis of CD4^+^FOXP3^+^ Tregs is observed in the GI mucosa of patients with active IBD in immunofluorescence studies [21]. Notably, IFX treatment inhibits apoptosis in Tregs and restores the number of FOXP3-expressing Tregs in the GI tract as well as in peripheral blood [18,19,21]. Further, induction of remission by IFX is positively correlated with the number of Tregs in the peripheral blood of patients with IBD [19]. Treatment with ADA also increased the numbers and percentages of peripheral blood CD4^+^FOXP3^+^ T cells [20]. Thus, expansion of GI and circulating Tregs is likely to contribute to the resolution of chronic inflammation in IBD after treatment with IFX or ADA.

TNFR2 expression on Tregs is required for their stabilization and homeostatic proliferation [22,23]. Nguyen et al. reported that the paradoxical enhancement of mTNF-α expression on monocytes after binding of ADA to mTNF-α induces Treg expansion through the interaction between TNFR2 and mTNF-α [20].

### 3.6. Alterations of Proinflammatory and Anti-Inflammatory Cytokine Production by Macrophages

The blockade of mTNF-α-mediated signaling pathways alters the profiles of cytokines produced by macrophages. ADA, IFX, and GLM carry the Fc portion of IgG1; thus, the interaction between the FcR of macrophages and DCs and the Fc of anti-TNF-α Abs induces cytokine production. Lymphocyte proliferation was inhibited in mixed lymphocyte reactions (MLRs) in the presence of ADA or IFX compared with the results obtained in the presence of ETN or control IgG [24]. The inhibition of lymphocyte proliferation required interaction between FcR-Fc because IFX F(ab’)2 fragment, which lacked Fc, did not suppress proliferation. Importantly, IFX binding to mTNF-α upregulated the expression of CD206 and IL-10, both of which are markers of macrophages with potent immunosuppressive activities. Thus, IFX can induce differentiation of immunoregulatory macrophages upon binding to mTNF-α [24].

Different from the induction of regulatory macrophages upon IFX binding to mTNF-α, the immune complex composed of sTNF-α and IFX induces proinflammatory rather than anti-inflammatory cytokine responses [25]. IL-6 production is enhanced by macrophages upon FcγRI activation by the IFX/sTNF-α complex. Thus, the development of regulatory macrophages by IFX may be controversial and requires further investigation.

## 4. Future Prospects and Conclusions

As summarized above, anti-TNF-α Abs used for IBD have diverse functions in terms of suppressing chronic inflammatory responses in the GI tract. Anti-TNF-α Abs exert immunoregulatory actions through binding to mTNF-α rather than sTNF-α. Induction of CDC, ADCC, and apoptosis by binding of anti-TNF-α Abs leads to a marked reduction in a wide variety of proinflammatory cytokines (IL-1β, IL-6, IL-12, IL-23, and TNF-α) in colitogenic macrophages and DCs expressing mTNF-α. Indeed, we experienced a case wherein UC was successfully treated with IFX even in the absence of enhanced TNF-α responses [26]. Induction of remission by IFX in this case was accompanied by markedly reduced IL-1β and IL-6 expression in the colonic mucosa without alterations in TNF-α expression [26]. We also reported a case of Behçet’s disease being successfully treated with IFX after failure with PSL [27]. Although both PSL and IFX reduced the mRNA expression of proinflammatory cytokines in the colonic mucosa, PSL failed to induce remission in this case. The successful induction of remission by IFX, but not PSL, may be explained by the enhanced mRNA expression of FOXP3 in the colonic mucosa after treatment with IFX. Thus, the careful observation and analysis of immune responses in clinical IBD cases provide mechanistic insights regarding how induction of remission can be achieved by anti-TNF-α Abs beyond the neutralization of TNF-α-mediated signaling pathways.

One question to be addressed in future studies is the effect of anti-TNF-α Abs on intestinal barrier function. Intestinal barrier disruption allows entry of intestinal microbiota into the lamina propria, thereby inducing proinflammatory cytokine responses by lamina propria macrophages and DCs [6]. Tight junction proteins such as occludins and claudins play critical roles in maintaining intestinal barrier integrity [6]. Anti-TNF-α Abs may restore intestinal barrier function through upregulation of occludins and claudins. In this regard, we experienced a case wherein CD was successfully treated with IFX after the failure of risankizumab, an IL-23p19 Ab [28]. Paradoxically, successful induction of remission by IFX was accompanied by marked reduction in claudin 1 and claudin 2 mRNA expression. Thus, reduction in claudin 1 and claudin 2 expression, which was observed after treatment with IFX but not after treatment with risankizumab, may have contributed to the downregulation of intestinal inflammation in this case. Further studies addressing the expression profiles of occludins and claudins before and after treatment with anti-TNF-α Abs for IBD are required to determine the effects of these biologics on intestinal barrier function.

Next-generation anti-TNF-α agents have been developed [29]. Ozoralizumab, a next-generation anti-TNF-α Ab, is composed of two anti-TNF-α-variable domain heavy chain (VHH) Abs fused with one human serum albumin VHH Ab. Ozoralizumab has a different structure from ADA, GLM, and IFX, and its small molecule size and albumin binding ability lead to rapid distribution in the inflamed joint lesions [29]. In addition, reduced adverse events are expected due to the lack of FcR-mediated inflammatory responses [29]. SOR102 is an oral bispecific inhibitor of TNF-α and IL-23p19 [30]. Dual inhibition of two major pathogenic cytokines by SOR102 is promising for the induction and maintenance of remission of IBD.

Concentrations in fecal calprotectin and serum C-reactive protein are widely used for the evaluation of anti-TNF-α therapeutic responses [31]. However, these markers do not always reflect the activity of gut inflammation. In this regard, expression of TNF-α in the GI tract may be useful for accurate evaluation of anti-TNF-α therapeutic responses. Atreya et al. developed a novel method in which TNF-α expression can be directly visualized in the GI mucosa of patients by confocal endoscopy after the topical administration of fluorochrome-labeled anti-TNF-α-Abs [32]. Thus, direct imaging of mucosal TNF-α expression could be achieved by a combination of fluorochrome-labeled anti-TNF-α-Abs and confocal endoscopy.

In conclusion, multiple modes of action beyond TNF-α neutralization contribute to the efficacy of anti-TNF-α Abs for patients with IBD. Biologics targeting IL-12/23p40 or IL-23p19, with fewer adverse events in comparison to IFX, are also available for patients with IBD [1,2]. Currently, whether anti-IL-12 or IL-23 Abs possess multifaceted functions, as verified for anti-TNF-α Abs, remains unclear. Anti-TNF-α Abs are the first biologics used for IBD treatment. The recent development and clinical efficacy of biologics targeting IL-12 and IL-23 may oblige IBD clinicians to consider the superiority of anti-IL-12 Ab or anti-IL-23 Ab over anti-TNF-α Abs in some cases. Notably, the well-established efficacy and safety of anti-TNF-α Abs are based on multiple modes of immunosuppression beyond neutralization of sTNF-α, and no studies have demonstrated the superiority of anti-IL-12 or anti-IL-23 Abs over anti-TNF-α Abs for the induction or maintenance of remission. Thus, the position of anti-TNF-α Abs among various biologics and small molecules that target pathogenic cytokines in IBD needs to be reconsidered. However, the duration of multiple modes of action beyond the neutralization of TNF-α has not been fully understood in ADA, GLM, or IFX. Furthermore, it remains unknown whether the next-generation TNF-α-blockers, including ozoralizumab and SOR102, share unique properties with conventional anti-TNF-α Abs. The accumulation of evidence regarding the mechanisms of action and correlation of clinical features will enable IBD clinicians to select the appropriate biologics for each case.

## Figures and Tables

**Table 1:** Multiple modes of action by anti-TNF-α antibodies used for IBD.

	ADA	GLM	IFX
Half life (days)	10–20	7–20	8–10
C max (μg/mL)	4.7	70.8	118
CDC	Yes	Yes	Yes
ADCC	Yes	Yes	Yes
Apoptosis of mTNF-α-expressing cells	Yes	Not examined	Yes
Apoptosis of lamina propria T cells	Yes	Not examined	Yes
Induction of regulatory T cells	Yes	Not examined	Yes
Induction of regulatory macrophages	Yes	Not examined	Yes

Abbreviations: ADA, adalimumab; ADCC, antibody-dependent cellular cytotoxicity; CDC, complement-dependent cytotoxicity; GLM, golimumab; IBD, inflammatory bowel disease; IFX, infliximab; mTNF-α, membrane-bound TNF-α.

## Data Availability

No new data were created or analyzed in this study. Data sharing is not applicable to this article.

## Abbreviations

The following abbreviations are used in this manuscript:

Ab	Antibody
ADA	Adalimumab
ADCC	Antibody-dependent cellular cytotoxicity
CD	Crohn’s disease
CDC	Complement-dependent cytotoxicity
DC	Dendritic cell
ETN	Etanercept
FcR	Fc receptor
FOXP3	Forkhead box p3
GI	Gastrointestinal
GLM	Golimumab
IBD	Inflammatory bowel disease
IFX	Infliximab
IL	Interleukin
MLR	Mixed lymphocyte reaction
NF-κB	Nuclear factor-κB
PBMC	Peripheral blood mononuclear cell
PI	Propidium iodide
PSL	Prednisolone
Th	T helper
TNFR	Tumor necrosis factor-α receptor
mTNF-α	Membrane-bound tumor necrosis factor-α
sTNF-α	Soluble tumor necrosis factor-α
Treg	Regulatory T cells
UC	Ulcerative colitis
VHH	Variable-domain heavy-chain
